# Steps Toward an Integrative Clinical Systems Psychology

**DOI:** 10.3389/fpsyg.2018.01616

**Published:** 2018-09-19

**Authors:** Felix Tretter, Henriette Löffler-Stastka

**Affiliations:** ^1^Bertalanffy Center for the Study of Systems Science, Vienna, Austria; ^2^Department of Psychoanalysis and Psychotherapy, and Teaching Center, Medical University of Vienna, Vienna, Austria

**Keywords:** cybernetics, synergetics, psychoanalysis, psychopathology, systems integration, systems science

## Abstract

Clinical fields of the “sciences of the mind” (psychotherapy, psychiatry, etc.) lack integrative conceptual frameworks that have explanatory power. Mainly descriptive-classificatory taxonomies like DSM dominate the field. New taxonomies such as Research Domain Criteria (RDoC) aim to collect scientific knowledge regarding “systems” for “processes” of the brain. These terms have a supradisciplinary” meaning if they are considered in context of Systems Science. This field emerges as a platform of theories like general systems theory, catastrophe theory, synergetics, chaos theory, etc. It provides a lot of abstract concepts, constructs, methods and models. We assume that these tools also enable theoretical integration in the diversified field of clinical practice in the sciences of the mind. Additionally, systems thinking in clinical psychology improves conceptual links to currently network-oriented neurobiology. However, clear definitions of systemic terms are necessary to emerge from their mainly metaphorical use. Here we revise mainly terms like “structure”, “process” and “dynamics” as they are used already in psychology, psychoanalysis, psychopathology and psychiatry in an ill-defined way. For instance, affective-cognitive structures like “life space” or “object representations” can be seen as products of mental processing. These structures, in turn, modulate dynamics of mental processes. Additionally, we suggest a coupled network concept of emotions and motivations as the main subsystem that modulates mental dynamics that results in a qualitative systemic model of the mind. Finally, we assume that a revisited systemic approach could improve interdisciplinary understanding of the mental.

## Introduction – Convergence in Plurality of Clinical Psychology

Nothing is as practical as a good theory

Kurt Lewin (1943)

Any disorder as a subject of clinical psychology, psychotherapy and psychiatry (here briefly: psycho-sciences) has a diverse history of conceptualisations, classifications and theoretical explanations. The common diagnose-related taxonomies like DSM 5 or ICD 11 are only of descriptive nature. Both taxonomies avoid causal interpretations by principle, disregarding (dynamic) psychopathology as well as neurobiology (Zachar and Kendler, 2007; Stein et al., 2010). Also quantifying rating scales as supplements for symptom checklists have no relevant explanatory value. This situation was criticized by NIMH, namely by Thomas Insel with his proposal for the Research Domain Criteria (RDoC): RDoC was developed as a conceptual framework that should be used by NIMH-funded psychiatric studies, especially if neurobiological research is conducted (Insel et al., 2010; NIMH, 2017). In this matrix - we discuss it again in the final section **–** the term “system” is used essentially and has a brain-related meaning (e.g., “negative valence system”, “systems for social processes”) but it is not specified in that context, it is some kind of a neurobiological “filler term” (Craver, 2007).

### Diversity of Approaches to Understand Drug Dependence

In order to give a clinically relevant *example* and to refer to a persistent public health problem *drug dependence* (including alcohol dependence) can demonstrate that an integrative view for understanding and treatment might be useful. First of all, the useful distinction of misuse and dependence in context of DSM 5 is subsumed under the general category “substance use disorders” (American Psychiatric Association [APA], 2013). Traditionally, in *clinical psychiatry* alcohol dependence is specified by symptoms that are related to alcohol consumption like craving, loss of control over consumption, neglect of other fields of life, symptoms at withdrawal or dose reduction, etc. As expected, DSM 5 shows nearly no effort to explain addiction unless one thinks of the high rate of *co-morbidity* (anxiety, depression, borderline personality disorder, etc.) that “drives” drinking but also could be “caused” by drinking (Atkins, 2014). In contrast, *psychoanalysis* (PA) assumes that drug consumption is a defense strategy to handle anxiety, anger and depression (“self-medication hypothesis”, Khantzian, 1987, 2003). In this view, psychoactive drugs are used because the *ego functions* are too weak to handle conflicts and because they compensate a *vulnerable self* that is experienced as a low and labile *self-esteem* (Dodes, 2009). Summarizing viewpoints of PA, addiction is the result of a disorder of *self-regulation*. In therapy PA relies mainly on (self-)exploration of mainly unconscious internalized representations of object relations (Kernberg, 1976, 1987; Krystal and Raskin, 1983; Joseph, 1985, 1989; Fonagy et al., 2002). In contrast, the causal model of addiction of *cognitive behavior therapy* (CBT) uses conscious descriptions (Marlatt and Gordon, 1985; Beck et al., 1990; Wright et al., 1993): in a stressful social situation, an addictive consumer thinks that he is disliked by others whereby this thoughts are enforced by underlying *dysfunctional cognitive schemata* (“I am a loser“). These schemata are self-related, and therefore a conceptual correspondence to the psychoanalytic concept exists, but CBT does not use explicitly the concept of an ego or a self, but implicitly of a *self-representation.* In therapy, the change of behavior, situation processing, stress coping, considering positive and negative consequences of drinking and abstinence, etc. are aimed.

Even if such constructs that are related to a concept of a self are similar, there is no agreement about it: in the framework of PA it is a structure of the mental apparatus, whereas in context of CBT it is the person’s recursive description of the person. Already some comparative discussion of the concept of the self occurs, however, an integrative process-oriented modeling is still missing (Kyrios et al., 2016). Integration of pathology of affective-motivational mechanisms discovered by PA (e.g., (Fonagy et al., 2002) and cognitive mechanisms – cascades of dysfunctional thinking – discovered by CBT, demands for our clinical example a comprehensive psychological model of addiction. Similar situations can be found in psychiatry of other mental disorders. Here we aim to constitute such a grounded framework.

A third important approach, mainly in context of neuropsychiatry, is brain-related (Koob et al., 2014; Volkow et al., 2016). Brain research has discovered many neuronal mechanisms as correlates of addiction, from prefrontal cortex to limbic system from neurons to the genes. This approach is driven by the hope to obtain sufficient “anti-addictive” medications although it turns now from a brain center-oriented paradigm to a (“systemic”) network view. At present, animal-based research also changes the terminology (“incentives”, “salience” system, etc.) that are partially represented in RDoC (Berridge, 2004). The neurobiological turn implicates a further gap in clinical psychology that is closely related to the brain mind problem (Kotchoubey et al., 2016).

Considering this kind of plurality of approaches in clinical practice of the sciences of the mind (Kendler, 2012), some questions are: How to integrate these views? Are “interdisciplinary” consensus conferences about terms like self, cognition or emotion helpful? Yes probably! However, also a “supradisciplinary” approach as provided by systems science seems to be fruitful.

### Preliminary Perspective of Systems Thinking

Systems thinking more or less is already implemented in the clinical psycho-sciences as very often the terms “structure”, “dynamics”, “equilibrium”, “complexity”, “self-organization”, “activation”, “inhibition”, “suppression”, “network”, etc. are used. The most prominent field where one finds such systems concepts is systemic family therapy (Winek, 2010).

We think that these terms are essential links for the often demanded conceptual supra-disciplinary “bridging” between different theories of the psycho-sciences. However, their use very often is only a fuzzy metaphorical way to describe some sometimes-hidden properties of the clients regarding their mental disorders. Namely this terminology often evokes the question “Structure, etc. – of what?” Although analogies are fruitful for theory building (Hofstadter and Sander, 2013), we think that they need to be “empiricized” stepwise. In context of systems science, these concepts appear as crucial descriptive-classificatory terms to build up stepwise a systemic, and therefore integrative, conceptual framework for the clinical psycho-sciences (Bunge, 1998; Tretter, 2005).

### Aim and Structure of the Paper

In this paper we focus on correspondence of “cognition” and “emotion” to terms like “structure”, and “dynamics”, but also “function”, “process”, “equilibrium” etc. We don’t intend a new conceptual taxonomy of the mental, especially of emotions and motives but a systemic version of some established concepts.

In this *first part* of our paper, we start now with the meta-theoretical - and in this way: *philosophical – basis* of explicit systems thinking, stressing the necessity of *qualitative systemic modeling* instead of usual *data-driven reasoning*. Several basic categories of psychology are mentioned and defined as systems thinking demands clear definitions of the elements of the respective “structured whole”. In line with this, we briefly mention some methodological and theoretical issues of *systems science* in the next section. In a *second part* of the paper we aim a higher precision of the use of the terms “structure” in the psycho-sciences, especially regarding concepts like “life space” or “representations of object relations”. In a next section, we explore the respective meaning of “dynamics”. In addition, we discuss fruitful applications of the *control loop paradigm* briefly, that enables a better integrative understanding of emotions and motivations and their interaction dynamics. Finally, we sketch an integrative conceptual systems model of the mind that allows for an integrative but differential and detailed description of mental structures, processes and their dynamics.

## Metatheoretical Aspects – Conceptual and Methodological Aspects of a Systems Psychology

### The Epistemic Cycle – Between “Empirics” and Theory

**FIGURE 1:**
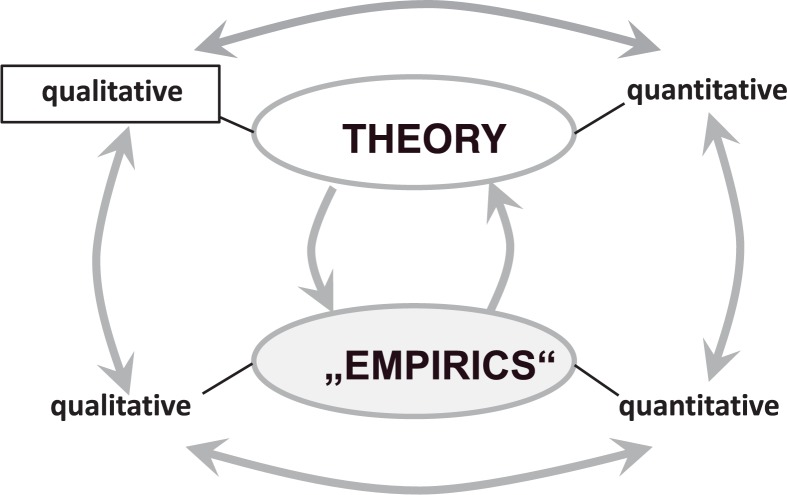
Interplay in research between theory and “empirics” and from a qualitative level to a quantitative (formalized) level of essential constructs, with focus on qualitative theory of the mental.

In this paper, we suppose to establish a *systemic framework of qualitative categories* to describe the structure and dynamics of the mind. This project seems to be opposing to current empiricism in the psycho-sciences and therefore it should be seen in context of general developments in the sciences: *history and philosophy of science* show that many sciences over decades exert a dominance of *experimental-empirical research* that are followed by periods with emphasis on *theoretical research*, also switching from *qualitative research to quantitative research* in *theory* and *empirical fields* (**Figure 1**). This cyclic epistemic long-term phenomenon can be captured by the term “epistemic cycle” (Bunge, 1998; Tretter, 2005). In case of “empiricism” of current behavioral neuroscience and psycho-sciences it is evident that already a huge amount of quantitative data already is available but there is a lack of a defined field of theoretical (neuro-) psychology that provides an appropriate integrative conceptual framework for quantitative and qualitative (or: semiquantitative) data and everyday observations and experiences. However, integrating quality and quantity of observations is important for “understanding” in practical clinical work.

Systems science could help to develop precise qualitative constructs and conceptual frameworks that can be measured by semi-quantitative (qualitative) scales (nominal and ordinary scales) and that are suited for a more comprehensive description of observations and for theory development. For this reason, the enforcement of theoretical research seems to be fruitful.

For instance, already in the 1970s the application of *mathematical catastrophe theory* in context of psychological issues provided an *integrative qualitative conceptual framework* for understanding both, a smooth and a sudden (non-linear) change of states of human behavior (Ginzburg and Gromov, 1979): anxiety under extreme pressure suddenly converts to aggression (Zeeman, 1976). Meanwhile, in context of dynamic systems theory already a huge amount of *quantitative psychological research* has been conducted mainly based on data of rating scales (Guastello et al., 2009; Schiepek et al., 2015). We believe that a wider conceptual re-visitation of the sciences of the mind might be useful to bridge gaps between systems theories and empirical research.

But first: what do we mean with “mind”?

### Epistemology – The Ontological “Reality” of the Construct “Mind”

The “mind” is the central epistemic object of the psycho-sciences. Here we understand the term “mind”, by mental “states” and “processes” and “structures” and “dynamics” and sometimes we use the terms “mental” and “psyche” with the same meaning. Basically, we claim that discussing psychological issues requires explicit epistemological considerations: The mind is conceived as a non-physical entity as it is an emerging organismal “disposition” that determines behavior, and before anything else it is a “construct” of the observer (Piaget, 1977; Maturana and Varela, 1987; Gergen, 1999; von Foerster, 2007; Levin, 2018). Finally, it is important to note that observations of other persons’ behavior (“objective” third person perspective) and observations by self-experience (subjective first-person perspective) have their specific heuristic benefits and limits. Therefore, in context of practical psychology they are complementary methods (pragmatic methodological dualism).

#### Consciousness

Furthermore, we assume that the central feature of the mental is “consciousness” (Scharfetter, 1980; Chalmers, 1996; Searle, 2004). In our view, it is a disposition to be awake, and aware of the world and of the subject itself. In contrast, in clinical context we see the respective disorders of consciousness like coma, coma vigil (persistent vegetative state) and (psychotic) disturbances of self-experience. Additionally, we have to admit unconscious (or preconscious) control of routinized behavioral patterns. They are seen here as a subset of mental states and processes as they correspond partially to the academic construct of subliminal, implicit mental processes as it was proven already in the 1970s by experimental psychological research in patients with brain injuries (Poppel et al., 1973; Weiskrantz et al., 1974). Also intuition as a vagely experienced but basic and parallel processing pathway at decisions is a mode of only implicit information processing (Kahneman et al., 2000; Gigerenzer, 2015). Another basic distinction of conscious processes is important: the experience that mental events can be classified as “reality-related” versus “phantasies”. For instance, plans and expectations are experienced as phantasies (or virtual realities). Additionally, a differentiation between the conscious and vivid experience of “reality” and the sleep-related “dreams” (and/or other states of altered consciousness) must be considered in a *multidimensional view of consciousness* (Scharfetter, 1980). Altogether, some of these concepts refer to the “known unknown” structure of human consciousness.

#### The Mind – Black Box or Functionally Structured?

For description of the functional structure of the mind, a lot of categories can be used, up to the infinite number of prosaic descriptions by literates. In line with this, already at the end of the 19th century, philosophers of mind have developed an extensive number of categories that were used to describe mental states and processes (Heil, 2004). In this field, also some taxonomies of the mind were designed and a variety of theories of action were proposed (Block, 1980; Block, 1981). Interestingly, at present the structure and function of emotions and motives is discussed in philosophy with the potential to connect studies of brain and mind toward an integrative behavioral neuroscience (Ben-Ze’ev, 2000; Hacker, 2004; Brüntrup and Schwartz, 2012). However, these categories are discussed insufficiently in the psycho-disciplines.

In the context of such conceptual diversity, already at the beginning of the 20th century Behaviorism claimed to study humans only regarding the observable behavior but disregarding their “inner” states and processes (Watson, 1930; Skinner, 1990). In this context it was assumed that the mind is a field of functionalistic-behavioral dispositions (Block, 1980; Bunge and Ardila, 1987; Searle et al., 1992; Levin, 2018). This black box conception with its input-output view and the image of men as a stimulus response machine promised a higher objectivity (i.e., intersubjectivity) of research results as research focussed more on the logical structure of experimentation and on the clarity of concepts that were used. In this way, animal experiments were also allowed to model human behavior. The stimulus response paradigm (S-R paradigm) determined psychological research since then and up to now. Pathological behavior is understood as the result of conditioning processes by stimulus coincidence and by operant conditioning that works as reward or punishment.

Functionalistic input-output analysis also allows for construction of hypothetical explanatory concepts that characterize the (sub)system under study as a function generator (or operator): it could be a receptor, effector, activator, inhibitor, amplifier, analyzer, synthesizer, a storage device, etc. This terminology is common in Computer Science and Electrical Engineering and was applied already in General Systems Theory (Miller, 1978; Tretter et al., 1982).

Although this kind of black box psychology increased scientific rigor, some researchers criticized the conceptual purism and induced the cognitive turn: Edward Tolman showed that rats learn more easy if they have the opportunity to internalize the spatial structure of the labyrinth to be learned by a hypothetical “mental map” (Tolman, 1948). These processes and their products are important intervening variables and were called “cognition”. Noam Chomsky also supported the idea of internal processing mechanisms, in his case regarding generators of language (Chomsky, 1959). A differentiated concept of cognition as a constructive process of internal representations of the world was finally introduced by Ulric Neisser in the 1970s with much success, so that the “cognitive turn” in the behaviouristic approach had an extreme boom that still holds today (Neisser, 1976).

In parallel, already at the end of the 1960s, the biologist and philosopher Ludwig von Bertalanffy criticized behaviorism and functionalism in psychology (von Bertalanffy, 1967): “Man is not a passive receiver of stimuli coming from an external world, but in a very concrete sense creates his universe.... Perception is not a passive mirroring of a world outside like a color photograph; rather, incoming informations are, by a creative act, organized into a universe”. This view of a constructive and not only representational mapping corresponded with the upcoming constructivistic turn and the concept of “autopoiesis” (Watzlawick et al., 1967; Bateson, 1972; Maturana and Varela, 1980). In line with this construct, the recurrence of activity via intra-organismic and extra-organismic feedback loops is constitutive for constructive conscious processes of the mind (von Holst and Mittelstaedt, 1950). This cyber-systemic concept corresponds to the phenomenological view that conceives the mind as embodied, embedded, extended and enacted (Pask, 1981; Clark and Chalmers, 1998; Noe, 2004, 2005).

It should be kept in mind that methodologically, the main distinction in the field of psychology can be seen between empiricistic, experimental and animal-based research and concept-oriented PA that is based on clinical observations: it is still not possible to model validly the feeling of guilt of a relapsed alcoholic by a rat experiment as some of these negatively affected humans would commit suicide! And also psychotherapy research shows the multitude of types of language to describe self-reports of patients (Shahar, 2018).

### The Mind as an Operationally Structured System – Some Essential Categories

In order to give a first view of essential elements of a systemic psychology, we root in methodological dualism, but start with the “objective” black box perspective understanding the mind as a structured system for *information processing in living systems*. In line with this, the mind is conceived as a system of multiple input-output devices (“black box of black boxes”). These basic functional operators exhibit feedbacks, divergence of outputs and convergence of inputs, etc. Such top–down driven conceptualizations of a modularity of the mind constitute several frameworks for mental states and processes (Fodor, 1983; Pöppel, 2010). They can also be seen in Artificial Intelligence (Minsky, 2006; Sun and Franklin, 2007). Therefore, we are zooming-in and at a first level of conceptual resolution we conceptualize the mental as macro-psychological multi-component system. Here we derive about a dozen key concepts of relevant mental operators that exhibit processes and states from terms as they are frequently used in chapters of textbooks of (general) psychology (Rohracher, 1988; Gross, 2010; Zimbardo et al., 2013; Tretter, 2016; Maderthaner, 2017). Also, clinical psychopathology relies on these categories (Jaspers, 1973, 2013; Broome et al., 2017). These operators can be distinguished but they are connected, tightly coupled but puffered, with their respective dynamics as it will be discussed later:

Receptive input functions (*perception*) and expressive output functions (*motor behavior*) are basic components of the stimulus-response perspective. They are the respective cross-sections between consciousness and the environment. They also constitute an environment-related and goal directed control loop as it is described in context of psychological action theory (Heckhausen and Heckhausen, 2010; Miller et al., 2013). Some perceptions are processed by *thinking* and stored by *memory*. Both processes are often categorized as “cognition” as a constructive “mapping” procedure. Output of thinking can be *plans of behavior* that function as goals and a guide for behavior. Memory in turn, as a feedforward process, provides a setting for perception that is experienced as *expectation.* Any misfit of expectation and perception can induce immediate resonance states that we call *emotions* such as surprise, anxiety, anger, sadness, etc. They are reactive but also spontaneously occurring states, can persist for hours, days or weeks and can influence perception, thinking, memory etc. They can evoke *motivations* (or desires, needs, etc.) as drives to behave, as for instance curiosity drives exploration of the environment. In this view, motivations are experienced as intentional, goal-directed drive states, that sometimes correspond to the issue of volition. From a clinical point of view, basic motivations encompass the desires to maximize pleasure, binding, orientation, control and self-value (Grawe, 2004): If they are not satisfied chronically they can evoke mental disorders.

We also think that the concepts of the experienced “I” (or: ego) and “self” (and self-image) are useful constructs, at least from a clinical point of view. In this context, the self is seen as a process-determining nucleus of the person (Jung, 1960). It is the reference object of the self-image (self-model, self-representation). The constructs “ego” and “self” correspond highly with the important construct “personality” that can be conceived as a system (Mischel and Shoda, 1995; DeYoung, 2015): these constructs describe the trans-temporal (relative) invariance of behavior dispositions. In consequence, we propose a differentiated but integrative network concept of the mental system in the final section of this paper.

However, that each of these mental operations mentioned above can be subdivided into sub-functions as any of the mentioned textbooks show.

There is another side of the mind, not processes and states but *mental contents*: basically, these are *images* of the world, the self and of the relations between them. They are often called “representations” or “affective-cognitive schemata” etc. and are mainly products of mental processing. In turn, as products and content of the mind they also modulate the mental processes and the behavior. We discuss them briefly in the second part of the paper the chapter on mental “structures”.

This set of conceptual building blocks of a meta-disciplinary modeling framework could integrate the diversity of views of psycho-disciplines such as analytic psychology, PA, cognitive psychology, biological psychology, etc., although these approaches exhibit significant differences in the definition of the subject, the concepts and conceptual frameworks, methodologies, theories and empirical and theoretical paradigms.

In this paper we try to identify bridges to be built to overcome some of these gaps that exist between the various psycho-sciences.

#### A Conceptual Dichotomy of the Mind – Cognition, Emotion and Interactions

For a first step, all the categories mentioned above can be up-scaled to two very basic and different mental sub-functions: cognition and emotions, where “cognition” is related to perception, but basically means thinking and memory and “emotions” – often also called “affects” – have an overlapping meaning with the term motivations. Although these mental entities are distinguishable phenomenologically, they are interconnected, coupled but buffered. This concept of “integrated differentiation” is connected to the historical cognition-emotion controversy in psychology that happened in the 1980s between Zajonc (1980) and Lazarus (1984) (see also Lai et al., 2012). The psychiatrist Luc Ciompi also *dichotomizes the mind* into emotions (affects) and cognition, a model that he calls affect-logic. Ciompi was showing for example, that focussing on cognitive disorders in schizophrenia might not help to understand the syndrome, whereas the role of emotional processes and states like anxiety, ambivalence, ambitendence, etc. seem to explain disorders of thought. We assume a *bidirectional causal relation* between cognition and emotion as we also acknowledge a top-down causation of emotions by (content) of cognitions. Referring to neurobiology it is still controversial, if (and how) the observable and obvious differences of neuronal circuitry of cognitive networks (e.g., cerebral cortex) and emotional networks (e.g., Amygdala) are functionally relevant for a concept discussion of psychological phenomena.

Regarding these distinctions, our understanding is as follows: Cognitions and emotions are phenomenologically different, can occur separately, but are connected and influence each other.

- Cognition means receiving, analyzing and synthesizing information. Cognitions are conscious but also have unconscious components therefore they are mainly an explicit (and implicit) kind of *information processing* that mostly uses symbols in context of a language (words, symbols etc.), which is a (second) signaling system. But cognition also happens without language (comp. language-free intelligence tests). Cognitions enable to construct concepts, they serve as structure generating processes, some products of that are parts of the content of the mental. These persisting products of cognitions (usually called “affective-cognitive schemata”) shape experiences and in this way also emotions. Cognitive processing (e.g., thinking), compared to emotions, has a faster dynamic and can be controlled by the person by concentration and attention and volition.

It should be mentioned here that RDoC provides a complex taxonomy of cognitive mechanisms that seems to be useful for more detailed systemic conceptualizations of the mind.

- Emotions are conscious experiences with deviations between pleasure and unpleasure, they constitute a valuating subsystem of the mental; they also obtain perceptual inputs that are only preliminarily processed. Emotions have somatic correlates that impose as modulated autonomic functions like sweat production, heart rate, skin temperature, skin conductance, blood pressure etc. Emotions (and motivations) act as drivers and brakes of the mental, respectively of cognitions. But emotions also activate or inhibit motivations that – as goal directed behavior – are drivers of behavior. Emotions can be controlled moderately, only motor expression can be controlled more easily.

Interestingly, referring to the matrix of the Research Domain Criteria of the NIMH (RDoC) emotions correspond to the “positive” and “negative valence systems”, but they are not represented in that taxonomy by theoretical considerations: for instance, it is unclear if sadness is only a “reaction to loss”, and if it could be also a reaction to non-occurrence of expected reward, we do not integrate the RDoC into our preliminary taxonomy of the mental.

Concerning a more precise definition of the *functional connectivity* between cognition and emotion, we want to refer to the *general theory-strategic problem* to construct models that *distinguish subsystems* but that have to admit that theses *subsystems are also connected*: some (social) systems theorists interested in the coupling of the mental world with the social world used the term (functional) “interpenetration” (Talcott Parsons in his General Systems Theory of social systems; Parsons, 1959), or “structural coupling” (Maturana, 2002) or the currently discussed notion of “resonance” (Rosa, 2016).

Summing up, in the second part of the paper, we concentrate on emotional and motivational aspects of the mind and ignore cognition a little bit, as cognitive science was the most successful research program for scientific psychology in the 20th and the 21st century, and as its results are widely known.

Aiming to match psychology and system science, we use this simple conceptual dichotomy of the mind as an affective-cognitive system to compare it with the abstract terms “structure” and “dynamics” of systems science.

### Reductive Bottom–Up and Top–Down Explanations – Neurobiology and Cultural Sciences

The current reduction of mental functions to brain functions encompasses a lot of well-known epistemological, methodological and conceptual inconsistencies (Nagel, 1974; Block, 1980; Chalmers, 1996; Noe, 2004, 2005; Craver, 2007). This we discussed recently in a multidisciplinary view of the problems of integration of the widely disciplinary diversified field of neuroscientific research (Kotchoubey et al., 2016): Mainly the qualia problem, known as the un-substitutability of the subjective first person perspective by the objective third person perspective, implies the need to construct a differentiated but integrated conception of the mind. We also stressed the fuzziness of empirical findings regarding structure-function relations: for instance, a multi-functionality of dopamine (reward, reward prediction error, acute psychosis) and also a multi-structural realization of reward functions (endorphines, oxytocin) converge to the problem of localization of function (e.g., more than 30 brain areas are involved in vision) (Felleman and Van Essen, 1991).

As one conclusion of our paper on methodological problems of integrative neuroscience, we suggested the development of a systemic view of the mind. This view needs not be justified by neurobiology, inspite of the current “network turn” in neurobiology. This implicates a methodological parallelism as it was seen already by the founder of General Systems Theory, von Bertalanffy (1967, pp. 100/101): “If both mental and behavioral or physiological events can be described by the same models, this means isomorphism between them”. Therefore, a systemic non-reductive multi-level approach might offer better options for integration (Miller, 1978; Henriques, 2004).

In opposition to reductive neurobiology (naturalism), reductive cultural sciences and phenomenology claim with good reasons to explain the processes and structure of the mind by cultural issues such as language, cultural values, social roles, etc. as the content of the mind (culturalism). In this context, it has to be considered that there is still a methodological gap between natural sciences and social sciences (or cultural sciences). Therefore, by zooming out of the person and focussing on the external sociocultural world we also might not find a complete explanation of the mental: theoretical connections between psychology and social and cultural sciences have to be discussed on various different levels (Luhmann, 1995; Kotchoubey et al., 2016; Schülein, 2016).

### Perspective

Epistemology in psychology shows that a diversified but integrative conceptual framework should be developed in relation to, but independently from neurobiological research (methodological dualism). Bridging naturalism and culturalism, a basic concept of the mind as a system that is based on the brain (embodied) and that is embedded and extended in and enacted with the environment could be developed (Clark and Chalmers, 1998). This phenomenological position of philosophy of mind corresponds closely to multi-level models of ecological psychology and human ecology (Tretter, 2008; Fuchs, 2018). In spite of this holistic view, we now focus on micro-psychological research issues. Finally, we demonstrate a systemic view that is based on the dichotomy of structures (cognition) and dynamics (emotions and motives). On this path, PA serves us with most interesting material as a theoretical reference framework (Schülein, 2016).

## Systems Science

In order to explicate the framework of systems science that we use for psychology, we give a sketch of this field. Currently, systemic studies, i.e., studies that are based on system theories and their methodological building blocks, are summarized by the term “systems science”, at least if one follows the International Society of Systems Science (Mobus and Kalton, 2015; International Society for the Systems Sciences, 2018). According to the diversity of the history of concepts that is rooted in cybernetics (Wiener, 1948) and general systems theory (von Bertalanffy, 1950, 1968, 1972) the term “cyber-systemic” is often used as an integrative term for systemic studies, models and practice. In line with this, we refer to “systems science”, not to any “systems theory” in particular.

Regarding living systems some qualitative theories were published (Miller, 1955, 1978): In the fields of psychological and psychiatric therapies, systems thinking and systemic intervention are already broadly established approaches, mainly by systemic family therapy (Winek, 2009). However, in this context, the systemic property to be (more or less) “connected to everything” means mainly that communications are interconnected between individual observers with individually constructed meanings that can be shared by the others. It also means that the individual meanings of communicative signals are learned from the social environment of the respective person (Watzlawick et al., 1967). Additionally, communication relies on implicit assumptions of the other, his or her intentionality, etc. in a recursive way – “I expect that you expect that I expect…”. In the systemic view, all these aspects are concerned with the social environment and with the embeddedness of humans to their context, also if they are mentally disordered (Bateson, 1972). However, in this paper we would like to focus on the mechanisms of the intrapersonal mental world that constructs the representations of the world, of the self, and of the interrelations between these components. With this aim, we briefly give a sketch of systems science and psychological applications.

### What Essentially Is “Systems Thinking”?

Like any other science, also systems science can be characterized by its subject (epistemic object), its conceptual constructs, significant conceptual frameworks and models, theories, empirical paradigms, etc. (Bunge, 1998).

In this respect, the epistemic object of systems science can be any system, regardless of its material realization. Significant concepts are “system”, “elements”, “component”, and more complex constructs like “feedback”, “self- organization”, etc. The specific methodology can be characterized as a supradisciplinary multi-level perspective and in line with this a procedure of context-sensitive analyses, modeling and computer simulations is significant. The essentially theoretical field of systems science is composed by components of communication theory, catastrophe theory, chaos theory, synergetics or complexity theory.

For better understanding (Zimmermann, 2015): The crucial term “system” is defined as a set of elements and a set of relations (structure and connectivity), and – in living systems – a boundary condition in relation to the environment should be identified (Hall and Fagen, 1956; Miller, 1978). Operationally closed systems (e.g., with feedback loops) are significant functional structures of living systems, conscious systems and social systems. In line with this definition, a system can be characterized simply by the term “structure” or by the popular expression “network” (nodes and edges) as it is a network with boundaries. Or, with other words: a living system is a network (or structure) with boundaries. Properties of systems are states (e.g., equilibrium, non-equilibrium) and processes, some of them have goal-directed functions as a subset of activities. Processes can be understood as changes of states. The properties of change of states – e.g., their speed and their intensity – are conceived as “dynamics”. Functional structures that determine processes are mechanisms. They provide mechanistic explanations, even in psychology (Craver, 2007; Bechtel, 2009).

These short working definitions of crucial concepts might be sufficient for our paper to combine some psychological issues with systems science. It must be said here also, that systems concepts very often are only used in a metaphorical way that does not enable real progress in understanding in psychology (Gelfand and Engelhart, 2012).

Systemic exploratory methodology basically implies to zoom into the micro-level of the subject of study, not forgetting the context and also to zoom out to the macro-level without forgetting the details. If we zoom out of the detailed consideration of elementary functions of the mind to a more holistic view we will refer to several holistic models that also will provide a diversified understanding of mental processes in context of clinical issues (Henriques, 2004). Regarding systemic methodology of modeling as it is relevant in this paper, it should be mentioned that we start with verbal models that explicate interactions and that in some cases are presented in graphs. Usually the next step should be a mathematical formalization of this hypothetical causal model but we don’t think this will really increase evidence here and therefore it should be reserved for a later step of discussions of modeling the mind. After the formalization, empirical data should be integrated and now it is possible to transform the model to a computer algebra system (e.g., Maple^®^, Matlab^®^, Mathematica^®^) for running simulations in order to explore the functional structure of the model by process analysis. This stepwise procedure was developed basically in the context of systems dynamics with regard to the development of the theoretically fruitful socio-ecological “world models” (Meadows et al., 1972; Sterman, 2000; Meadows and Wright, 2015). This procedure has already been applied within exploratory computer simulations several times for psychiatric disorders (Schiepek et al., 1992; An der Heiden et al., 1998; Tretter and Scherer, 2006). Interestingly, an integrative systems dynamics model of depression was published recently (Wittenborn et al., 2016).

### Some Cyber-Systemic Models and Theories

**FIGURE 2:**
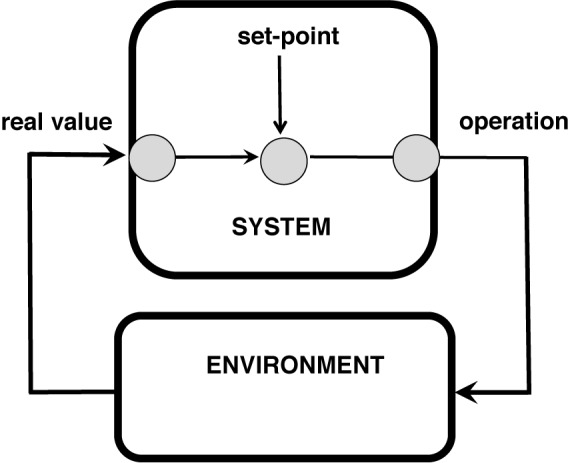
Control loop paradigm (e.g., heating regulation).

One of the most useful conceptual framework of systems science is the control loop model (**Figure 2**): An operator compares (or computes) the value of a set point with the received actual (or: real) value of the state of the environment (e.g., temperature). In case of lower actual value than the set point an effector (e.g., the heating) is activated and a regulatory action takes place (**Figure 2**). In psychology, the study of biological desires and needs (or motivations) was successfully related very early on the control loop model as it will be discussed below (Carver and Scheier, 2012). The most influential application of the control loop metaphor constituted the basic psychological action theory like the T-O-T-E concept (test, operate, test, exit) of goal-directed behavior (Miller et al., 2013). It also fits the structure model of Sigmund Freud as it will be clarified later. A control loop concept was also used by Norbert Bischof for modeling binding theory as it is discussed later (Bischof, 1975).

**FIGURE 3:**
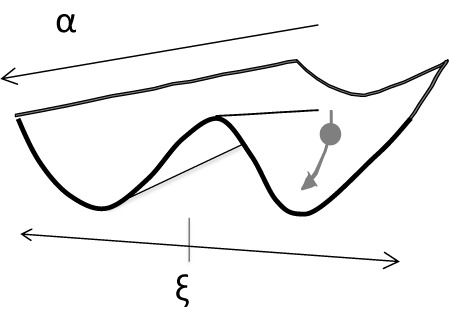
Potential landscape and ball as symbol for the system’s state in context of synergetics (α: control parameter, ξ: order parameter).

One heuristically very useful conceptual framework (or even theory) is synergetics that helps to conceptualize any system by two basic mechanisms: one regarding order parameters (structure) and the other regarding control parameters (Haken, 1977). This theory was validated by a theory of the origin of laser light. Both, the energy-relevant control parameter α (e.g., stimulating light) and the structure-relevant order parameter ξ (e.g., degree of synchrony of activation of electrons) determine the dynamics of the system (e.g., emission of highly synchronized light radiation). The regions of stability and instability in state space are called “attractors” and “repellors”. They can be symbolized by a polymorphic potential landscape where – in metaphorical use – the system’s state is represented as a ball that is moving according to the shape of the landscape (**Figure 3**). This landscape metaphor visualizes also the power of “attractors” (valleys) and “repellors” (hills) that determine the course of the system’s state (trajectory). Divergence of trajectories depends on the different relations of α to ξ: if α has a high value in our example a bistable state landscape occurs and a switch from one order to the other can hardly be realized. In case of a low energetic level of α, any weak perturbation can switch the state of the system easily. If the control parameter increases, the movement to one of the two valleys can be hampered. In this picture of a landscape, a ball can roll to different spots depending on the incoming forces and the shape of the respective spot of the landscape (**Figure 3**). In consequence, this model allows one to understand intuitively self organization to be constituted by these two factors. This synergetic view of dynamic systems was already used fruitfully in context of clinical psychology and therapy research (Schiepek et al., 2017). Later, we apply this concept metaphorically to psychoanalytic object relations theory.

Also, catastrophe theory, which is a mathematical concept that integrates linear and non-linear behavior as it was mentioned before, was applied in clinical outcome research in alcoholism (Witkiewitz et al., 2007). The well-known chaos theory that can identify chaotic systems states that exhibit random-like patterns is formalized by non-linear differential equations and shows that systems can autonomously generate irregular and regular patterns of state trajectories. For exploring clinical courses of alcoholism we have already applied chaos theory (An der Heiden et al., 1998).

### The Network Paradigm

Finally, and at present, the very old concept of network is substituting and supplementing the term system. Already since the 1960s, many computational models of the brain were constructed in context of “neuronal network modeling”. These models are based on hundreds of conceptual excitatory and inhibitory interacting neurons. Neuronal networks as a way of modeling networks have succeeded in theoretical understanding of various brain phenomena such as the occurrence of gamma oscillations (Liljenström, 2010).

Lately in the field of Molecular Systems Biology (Kitano, 2002), with its high-throughput molecular biological technologies and huge data sets, graph theoretical network approaches were used to identify patterns in complexity. These graph-theoretical analytic tools for data analysis are a next processing step after multivariate statistical analysis in order to identify functional proximity of active elements in the network (e.g., expressing genes). Also in context of biological psychiatry a huge increase of in-detail knowledge of brain chemistry came up by these high-throughput technologies and by graph-theoretical analytical tools (Tretter et al., 2010; Tretter and Gebicke-Haerter, 2012). This led to the “omics”-related “network turn” in neuropsychiatry that aims to inventory the molecular structure of the genome, the transcriptome, the proeteome and the metabolome. Graph theory as method of formal data analysis of complex data sets is also used in context of psychopathology referred as “network psychopathology” (Stein and Ludik, 1998; Borsboom and Cramer, 2013; McNally, 2016; Borsboom, 2017). In consequence, a parallel “psychomics”-turn appears to be fruitful in order to develop a comprehensive view on the multiple processes and states of the mind that can be conceived as a system (or network) of perceptions, thinking operations, memory functions, emotional states, motivational states, etc. as these single operations (and sub-operations) are interrelated.

**FIGURE 4:**
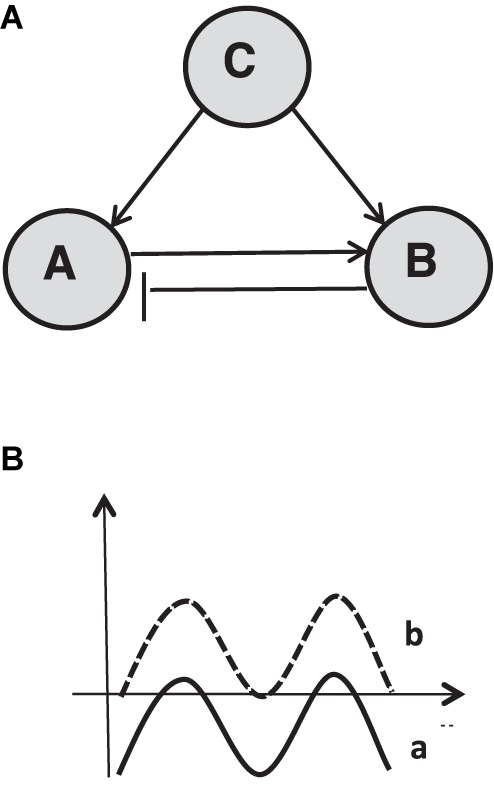
Network of oscillatory behaving activator-inhibitor module. **(A)** Activator A and inhibitor B are driven by activator C. **(B)** Non-linear behavior pattern generated by this module (oscillation). Activity of unit A **(B)** can be changed (e.g., elevated) by increase of activity of C.

Additionally, it should be discussed here briefly, that network modeling results in nice looking complex graphs with colored dots and lines. Those complex structure-oriented graphs exceed human comprehension and must be supplemented by numerical indicators for properties of the networks (centrality, clustering, etc.). For this reason and for practical use, simple models with a modular structure might be a good heuristic tool. They allow a *bottom-up understanding* of complex graphs and their constitution if it is assumed that any node of a network could be interpreted as the representation of another network (subsystem) and vice versa. In this simplification, one can start with an elementary two-component system with elements A and B and with two reciprocal connections with different modes of action: A can activate B, whereas B inhibits A, which results in an oscillatory behavior. Also, A can activate B and B can activate A, so that an escalatory process structure is given. Finally, A can inhibit B whereas B also inhibits A with the consequence that one of the two is the winner with polarized trajectories. By conceptual aggregation of multi-unit systems, one could qualitatively understand the dynamics of the system by appropriate exploratory computer simulations. These main dyadic schematics enable to reconstruct complex graphs of complex systems. This was shown for molecular networks by systems biologist Uri Alon with his concept of elementary substructures that he calls “motifs” and that also allow to simulate processes that are determined by these modules (Alon, 2007) (**Figure 4**).

### Preliminary Thoughts on Systems Psychology – The Mind as a System

In the view of general systems theory, the mind is a (structured) function subsystem of the organism (von Bertalanffy, 1968). It is treated similar to the “conceptual nervous system” as it was described by Hebb (1955) who used this term for a selection of concepts of the functional structure of the brain for psychological purposes. Any transformation of the concept of the mind to a concept of a computing machine also has severe limitations, although discussions in computational psychology (Montague et al., 2012) and computational psychiatry (Friston, 2012) bring up some refreshing impulses for new perspectives. Computational neurophilosophy as it was designed by Patricia and Paul Curchland, is an approach that should substitute methodologically and conceptually simple “folk psychology” (Churchland, 2007). Nevertheless, this approach is still in its infancies and does not help yet in fields of clinical applications.

In our view, the mind is a bounded field of processes and states that can be related to the brain but it “is” not the brain in a simple understanding of the term. Our view corresponds strongly to a process ontology. For reasons of brevity of our analysis we have to concentrate on the functional structure of the mind as an information processing system.

### Perspectives

Applications of constructs of systems science in the field of psychology can be focused on concepts like “structure” or “dynamics” as will be done below. Several models – e.g., control loop concepts, synergetics, chaos theory – can be used to re-conceptualize essential findings in (clinical) psychology. Probably a graph theoretical oriented network approach might be the most integrative theoretical language as it can combine empirical data analysis with structure-functional systems analysis very nicely.

## Mental “Structures” – Global Models of Conditions and Effects of Affective-Cognitive Schemata

It is important to identify mental processes as dynamics and their mental products as structures although they are tightly connected in mental functions. Here we focus on the notion of “structure” but it should be kept in mind that structure also exhibits long-term dynamics and that dynamics also exhibits a (process) structure. The connectedness of mental processes and mental products is traditionally described as the “intentionality” of the mind (Brentano et al., 1973). In line with these clarifications, it should be kept in mind that in the context of the psycho-sciences the term “structure” is very ill-defined: cognitions, personality, etc. are epistemic reference objects for this term. Even processes exhibit structures, for instance in time series of measurements of the chaotic ups and downs of emotions.

In our view, the mental products are structures and content of the mind (representations). As “structured structures” by explicit inter-level relations of these structures, for instance by reference to symbolic representations (e.g., language system) they are also semantic networks. In consequence, we use the term structure here when referring to the “content” of consciousness as “affective-cognitive schemata” or “affective-cognitive models” or “representations”. These affective-cognitive schemata represent more or less conscious images of the world, the self and the world-relations of the self. They operate as reference frames for new experiences by shaping perceptions, expectations, thinking, memory, decision, etc. They are closely related to the linguistic subsystem that we relate to cognition and therefore neglect in this paper: we focus on emotional-motivational processes that we distinguish from cognitive processes. In consequence, both kinds of processes co-produce explicit and implicit affective-cognitive schemata. It is supposed here that cognitive structures have an emotional-motivational loading. This model also corresponds to the concept of “structure” in context of traditional theories of cognitive balances and cognitive dissonances (Heider, 1946; Festinger, 1957). In line with this, we think that graph theoretically oriented theory of balance and dissonance (Cartwright and Harary, 1956) still could be very fruitful for empirical research in psychiatry (Tretter, 2013).

However, here we focus only briefly on the traditional concept of “life space” by Lewin (1951) and on the theory of representations in context of PA (Kernberg, 1976). Both approaches indicate the importance for clinical work to explore the structure of the outer world regarding the structure of the inner world and vice versa.

### Life Space in Context of Field Psychology

**FIGURE 5:**
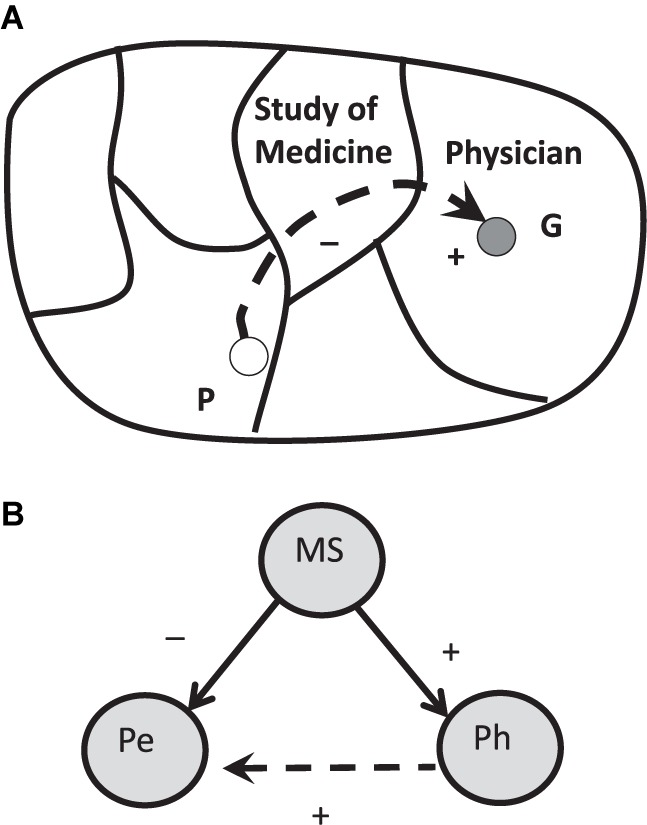
The structure of the life space with the topological location of the person (P, Pe) and the intended goal (G, physician), that has a high valence for the person to achieve, but some fields are barriers and puffer the goal area. For instance, to become a doctor, one has to pass the time-consuming barrier of the medicine study (Lewin, 1951). Topology **(A)** and network representation **(B)**.

The “life space” is a conceptual model in context of field psychology developed by Kurt Lewin that denotes the internal representation of the psychological environment of the person. Interestingly, Lewin used mathematical concepts from topology and vector mathematics to define psychological fields and vectors that should indicate theoretical assumptions regarding personal drives and environmental affordances of a person in their situation (Gibson, 1977). The life space is a topological concept that represents experiences as relations to their components. It consists to a large proportion of fictional worlds such as future states of compartments of the life space (Lewin et al., 1936). Lewin suggested that humans are always on the way (vectors) in a subjectively experienced and segmented representation of the world. For instance, if a person (P) wants to become a doctor (goal) she must pass the study of medicine with many exams that are barriers on the trajectory to become a doctor (**Figure 5A**). Generally, the sub-fields of the life space have emotional-motivational loadings that were conceptualized as the “valence” of the environment. The intended sector (goal) is surrounded by other sections of the life space that are separated by boundaries so that the person cannot reach them immediately. In our example, the field between the present situation and the intended situation is the study of medicine. As developmental psychology shows, the framework of the life space allows to construct a structured model of the experience of the situated person that in turn can be understood as a structure that modulates subjective information processing of the current state of the environment (Barker, 1968; Bronfenbrenner, 1979). In that respect, Lewin founded not only (mathematical) systemic psychology but also ecological psychology that is interested in the person in the world as a situated subject.

This concept captures the basic dynamics of life and its “directedness” toward goals by their “valence” (or attraction) to act. This concept of valence was differentiated later by James J. Gibson by the concept of “affordances” that is constitutional in context of ecological psychology (Gibson, 1979). Both concepts indicate the affinity of the person toward elements of her environment. For future quantitative research, these field concepts should be translated into network concepts (**Figure 5B**).

### The Object Relations Theory

**FIGURE 6:**
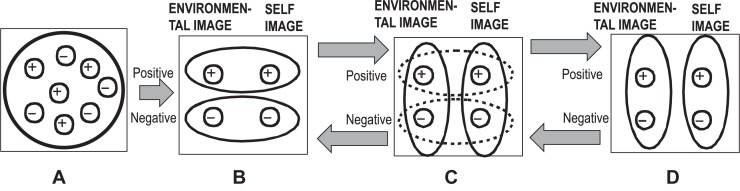
**(A)** Undifferentiated matrix, **(B)** early bipolar matrix, **(C)** transitional matrix, and **(D)** differentiated adult matrix. Visualization of the core concept of object relations theory as a matrix: the configuration of the self in context of objects, with positively and negatively experienced properties (Kernberg, 1976). Remarks: The term “object representation” is translated here for reasons of general applicability into “environmental image” and also “self-representation” is translated to “self-image”.

The object relations theory as it is currently proposed in context of PA by Kernberg (1976), is another important structure-dynamic view on the representational level of the mind. It is also a fruitful model as it focuses on the representations of the self, the world and the relations between the self and the world. These images are partitioned into two basic value fields as they are emotionally loaded by “good” emotions and “bad” emotions. These representations can be conceived as affective-cognitive schemata as they are understood in context of cognitive psychology as it was mentioned previously. They exhibit a basic two-dimensional structure that frames the individual developmental experiences in childhood: the first dimension represents the positive or negative emotional valence and the other dimension represents the topological-psychological proximity of the self to its environment (“objects”). During development, the early unstructured emotionally dichotomized experience matrix with a mix of good and bad experiences (**Figure 6A**) is transformed into a dual matrix with polarized good and bad experiences without a sufficient differentiation between self and the others (**Figures 6B,C**). Later in adolescence, this type of matrix is successfully integrated into a new formation that differentiates clearly and stable between the self and the other (**Figure 6D**).

**FIGURE 7:**
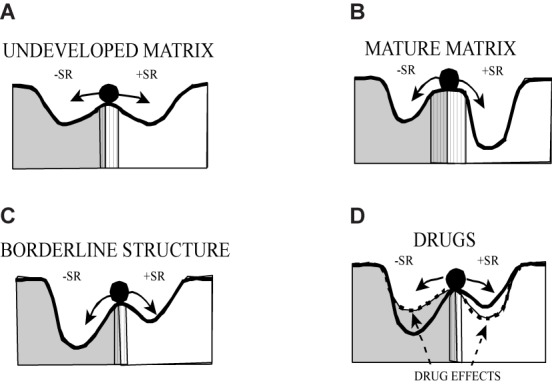
Borderline psychodynamics mapped to a landscape of object related self-representation: Emotionally positive components and negatively loaded components of the self-image have differently strong attraction that is indicated by the deepness and steepness of the valley of the potential landscape. The respective shape determines the stability of the current mental state indicated by the black ball. During development of the person the structure of the landscape becomes more accurate **(A,B)**. An intermediate level of development is characteristic for borderline personality structure **(C)**. In this case, by drug consumption older and therefore less structured “landscapes” can arise during intoxication with confused psychotic states of experience **(D)**. –SR, negative self-representation; +SR, positive self-representation [adapted from Tretter (2011)]. Reproduction with permission of Schattauer Publisher.

Hypothetically, in a systemic view the reconfiguration of this basic affective-cognitive schemata (or matrix) is optimized in the unconscious domain by principles of self-organization. In line with this, the categorical concepts of object relations representation theory were transformed to a semi-quantitative model of a potential landscape in order to emphasize the peculiar shape of the dynamics of borderline-caused information processing (**Figure 7**). The positive and negative experiences are depicted as basins of attraction, separated by a repellor-like wall of different shape. This shape of the surface determines the mode of information processing: an easy shift from positive to negative emotions and vice versa is possible by the low structuration of the landscape in early stages of development (undeveloped matrix) – single negative experiences easily induce a generalized state of negativity. This is a feature of emotional instability.

### Perspectives

For a future task, the model of the representations of object relations could be diversified in respect to the life space model, but it should also be “dynamized” in correspondence to the vectorial thinking of Kurt Lewin. Both concepts could be integrated by a graph-theoretically oriented conception of the representational matrix. By this method, not only general nomothetical models could be developed but also individualized idiographic models. This development would provide a more valid and coherent conception of affective-cognitive dynamics.

## Mental “Dynamics” – Global Models in Clinical Research and Practice

In this section, we emphasize mental dynamics that is driven by emotions and motivations. In this view, PA was probably the first school in clinical work that focused on the pathogenetic power of emotions and drives. Also the psychopathologist Werner Janzarik (Janzarik, 1988) systematized properties and interactions between structural and dynamic elements of the mind with his concept of a “structural dynamics” of psychiatric diseases. Finally, the psychoanalyst and psychiatrist Luc Ciompi has developed a systems theory of mental disorders – namely of schizophrenia – that emphasizes the function of emotions (“affects”) in respect to cognition. He called his theory “affect-logic” (Ciompi, 1997). Here we explore these approaches briefly in addition to above mentioned positions.

### Systemic Views in Psychoanalysis and Psychodynamics

As Sigmund Freud was summarizing in his “Outline” in 1938, he suggested already in about 1900 that mental process should be examined from topographic, dynamic and economic viewpoints (Freud and Strachey, 1949). Theoretically he was under the influence of physics of dynamic processes of that time (thermodynamics, electrodynamics, mechanics). Some of his basic concepts that are relevant for a systemic view are:

^∗^ The topographic model: This concept primarily distinguishes conscious and unconscious mental states and processes as it was described at the beginning of this paper.

^∗^ The psychic energy (libido): This concept describes a mental energy with an unspecified ontology. Therefore, it is also a source of misunderstandings, reinterpretations, etc. However, this concept indicating some “power supply” for the mind corresponds well to the control parameter of synergetics that changes also the level of order. Also currently in neuropsychiatry, the concept of “free energy” of the brain published by Karl Friston has a rich heuristic value in understanding structural dynamics and emergence of mental phenomena (Friston, 2010; Connolly and van Deventer, 2017). It is a fruitful metaphorical import of a concept of thermodynamics into neuropsychiatry referring to the organismic process of mapping the chaotic external world into an ordered internal map of the world on the basis of an action cycle.

But still, for pure (substrate-free) psychology the question remains is: free energy of what? Of probability distribution of (immaterial) beliefs and expectations? But how can we measure the beliefs? Probabilistic measures are o.k. in principle, but what are the instruments to obtain appropriate psychological data? There are several hidden epistemological problems with this concept.

^∗^ The structural model: This model distinguishes the id, the ego and the superego as relevant components of human information processing of the inner and outer world. The relations between these elements of the “psychic apparatus” represent the dynamics as the id is supposed to activate the ego by desires and emotions. The ego also has to refer to the superego that signals inhibitions (“You should not do it!”) or activations (“You shall do it!”). Both potentially opposing inputs to the ego have to be coordinated by the ego regarding the demands, frictions and options of the actual external world. The ego is also coupled with unconscious defense mechanisms that by filter operations protect the conscious ego if the pressure or tension of internal and external conflicts – superego versus id or appetence-appetence, approach-avoidance, etc. – becomes high. In the case of chronic conflicts, clinical syndromes can occur.

In order to translate the structure model into a diagrammatic cyber-systemic model, blocks have to be distinguished that are reconnected by arrows although Freud did not demarcate the three instances in his diagrams. By a block diagram, the structural model can be transformed into three coupled control loops – the demands of the environment are related to actualized demands of the id and to demands of the superego. This means that two intrapsychic control loops with potentially two antagonistic set points converge to the ego as the central regulator. These loops are connected and overlapping. In case of conflicts, defense mechanisms are activated by the ego, whereas in case of congruence, the behavior is congruent with the environmental demands.

**FIGURE 8:**
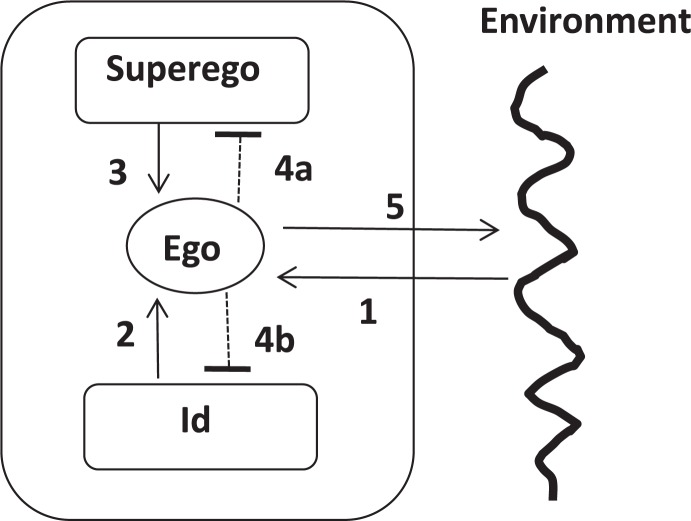
Block diagram of the implicit cyber-systemic structure of the structure model.

A simple process description explains the scheme (**Figure 8**): an environmental stimulus (1) that (unconsciously) evokes a desire (2) is received by the ego. In parallel, a demand from the superego evokes a pressure to the ego (3) that in consequence can suppress this demand (4a) or the intensity of the desire (4b). Finally, a certain behavioral reaction occurs (5). If a conflict between demands of the id (aggression!) versus the demands of the superego (be polite!) persists, neurotic symptoms (e.g., generalized anxiety) can develop.

Several other translations of PA into systems concepts appear to be fruitful, as it was claimed by psychoanalytic authors already (Galatzer-Levy, 1995; Seligman, 2005).

### Structure Dynamics in Psychopathology

In line with classical psychopathology, that was founded by Karl Jaspers in the early 20th century (Jaspers, 1973, 2013), Werner Janzarik developed a framework that he called “structure-dynamics” and that aimed to describe psychiatric syndromes in categories of structures, states and processes (Janzarik, 1988). He dichotomizes the mental domain and distinguishes mainly the terms structure and dynamics that characterize the mental system as a processor that can process information with low coherence in case of mental disorders. He describes syndromes in terms of properties of the (cognitive) structure and the (emotional-motivational) dynamics:

^∗^ “Structure” is defined as the internal representations of the world that are also loaded with emotional values. Deformations of the structure are weak structural changes that are compatible with subclinical everyday behavior. In case of pathological change, the structure shows autonomizations or even disintegrations as they can be observed in psychotic patients. For persons with obsessive disorders and also for delusions a change-resistent fixation of the structure is assumed. In other mental disorders destructurations and even simplifications of the structure are prominent.

^∗^ “Dynamics” is understood as life power, desire, drive, wanting, feelings and moods. Changes of dynamics in everyday life are called excursions (German: “Auslenkungen”). In pathological cases they appear as stronger and persistent derailments. A manic syndrome is seen as a dynamic expansion whereas in case of depression the derailment is classified as a dynamic restriction. Also distorsions (German: Verwerfungen) are properties that should classify different types of dynamics. The term discontinuity stands for disruptions of thinking in schizophrenic patients – and dynamic insufficiency classifies thinking abnormalities in chronic schizophrenia.

Although the concept of structural dynamics is very well respected in German psychiatry, the measurement of the variables like “derailments of dynamics” is unspecified. The challenge remains to transform these concepts into a systemic view: a transformation of “structure” into a graph theoretical view and “dynamics” into time-intensity diagrams could only be realized by close cooperation between systems science and specialized psychopathologists.

### Affect-Logic of Mental Disorders

A basic dual structure of the mind, similar to Janzarik, was also assumed by the psychoanalyst and psychiatrist Luc Ciompi who used concepts of systems science for explanatory purposes. His model is based on research in schizophrenic patients regarding their different long-term dynamics of symptoms – in some patients they disappeared after a schizophrenic episode or reoccurred after a while or in other cases, a residual dysfunction persisted (Ciompi, 1980). In some patients the affective disorders of schizophrenic patients were so obvious that he hypothesized that affective forces determine the cognitive domain. For this concept he coined the term “affect-logic” (Ciompi, 1997). He distinguishes interest, fear, anger, sadness and joy as basic affects that are involved in the formation of cognitive processing and cognitive structures as products. In line with this, he emphasizes the fractal similarity of affect dynamics between the micro-level and the macro-level of time scales.

### Control Loop With Expectation and Perception as Inducer of Emotions

**FIGURE 9:**
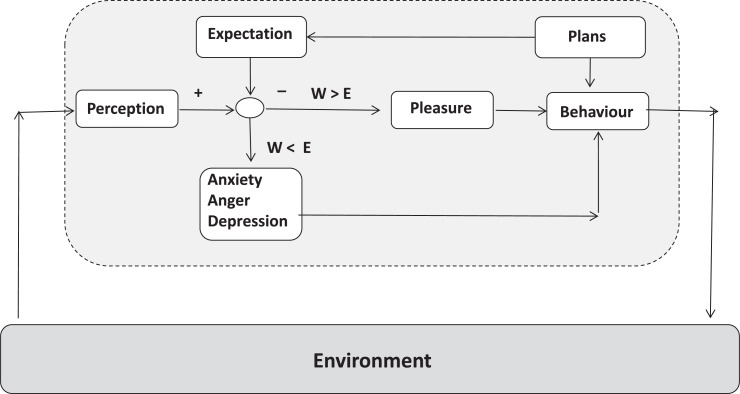
The control loop model applied for explanation of the occurrence of pleasure and unpleasure (anxiety, aggression, sadness) by the congruence (or incongruence) of expectation compared with perception. Also, expected versus perceived outcome of planned action can induce emotions according to this circuit.

In context of a general control loop model of the situated mind, emotions can be derived from the structure of cognitions (**Figure 9**). The relation between expected situation and perceived situation can explain the occurrence of emotions – if the expectations of positive events are higher than reality, anger, anxiety or sadness will arise. In case of lower expectations than reality presents, joy will come up. In principle, this circuit already was constructed by Leon Festinger who studied the relation between expectations and perceptions and by John Dollard and Neal E. Miller in context of the frustration theory of aggression (Dollard et al., 1939; Festinger, 1957). After some decades of research activities guided by this paradigm, a silent period followed, but today the crucial difference between expected (or predicted) stimulus and perceived stimulus is debated again by the prediction error paradigm (Montague et al., 2012; Sun, 2008).

**FIGURE 10:**
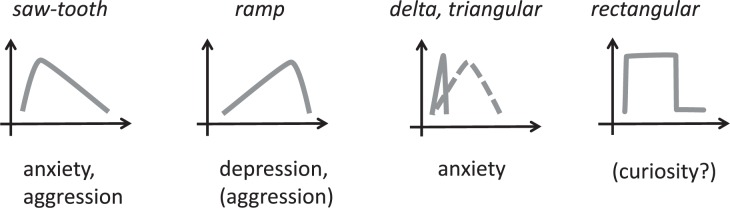
Hypothetical emotion-specific intensity-time profile that could characterize the dynamics of emotions. Saw tooth, ramp, delta/triangular and rectangular functions can be distinguished in context of S-R oriented phenomenological systems analysis. Some emotions appear to fit specifically to these profiles.

In line with these models, it is important in clinical practice to work on the individual relation between goals and/or expectations and experienced “reality” in order to develop a preventive stress coping strategy that aims to adjust expectations (and planning) to “reality” (**Figure 10**). To give a clinical example: change-motivated alcoholics, for further life, plan to drink no alcohol anymore. This evokes a latent but high tension and stress level. In case of a relapse an extensive negative emotion will arise that even can lead to suicide attempts as clinical experience shows. In contrast, if abstinence-oriented alcoholics only every morning, plan to drink nothing a much more life-cycle related planning with small control cycles is established. This evokes a lower level of tension. And even if a relapse happens, the drinking person can start the next day with his commitment to the daily abstinence goal.

**FIGURE 11:**
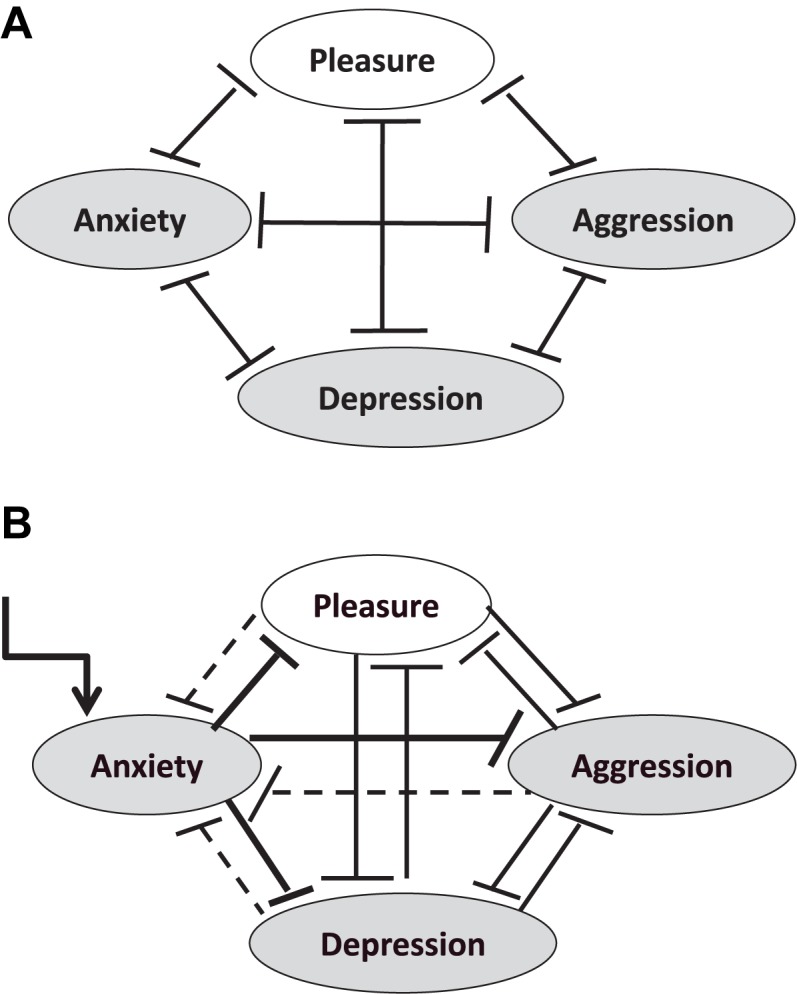
Hypothetical network of basic emotions with reciprocal inhibitions. **(A)** Balanced network with reciprocal inhibitions. Also co-activation can occur by dis-inhibitory feedbacks via double inhibition. For simplification bidirectional arrows are used. **(B)** Anxiety-specific stimulation evokes strong multilateral inhibitions that in consequence enforce anxiety by weaker inhibitory feedback. Unidirectional arrows are used here.

### Perspectives

Regarding these attempts to understand the interaction between cognition and emotions (and motives), it seems to be promising to combine these approaches in a qualitative integrative systemic framework. With this aim, we look closer to the micro-level of mental processes, namely emotions and motives, and check their structure and dynamics, sometimes referring to PA.

## Systemic Network Models of Micro-Psychology

Here we focus on emotions (or synonymously used here: affects) and motives (or desires, needs) as subsystems with driving and braking forces upon cognitive processes. In this view, emotions and motives determine mental “dynamics”. Several taxonomies for emotions and motives are used, however, it seems to be very hard to bridge their differences. Even the number of basic emotions can be seen as nearly unlimited and although everyday semantics of words that are used in different cultures were explored already, in consequence no consensual general taxonomy could be established yet. For instance, the pathogenetic relevance of basic emotions like disgust or cognitive feelings like curiosity or self-related feelings like shame or guilt are hard to be connected between different psycho-disciplines. For this reason, we focus only on a few emotions and motives.

### Structure of the Emotional System

At first it must be admitted that emotions are distinct mental conditions, not only side-effects of cognitions – they are coupled with but buffered from cognitions: “content-free” emotions like anxiety can occur as Zajonc claimed (Zajonc, 1980). One also has to consider the diversity of terminology: affects, emotions, feelings, mood, etc. For reasons of simplicity, here we use the term “emotion” and sometimes without a semantic differentiation, “affect”. This diversity must be considered if taxonomies are used: 2, 3, or 6 main or primary emotions are distinguished in literature (Plutchik, 1991; Ekman, 1992; Panksepp, 1998). An integration between these taxonomies is not possible as some basic emotions such as sadness are not depicted in Ekman’s and Panksepp’s taxonomy and Plutchik does not classify anger as a primary emotion, etc. Also emotions like guilt or shame have no conceptual congruence between these frameworks.

For more detailed description, each emotion could represented by a bipolar multi-dimensional property space (Wundt, 1898): Tension versus relaxation, pleasure versus displeasure; excitement versus calmness. Also their dynamics can be classified: fast versus slowly occurring and fast versus slowly disappearing emotions, etc. (Larsen, 2000; Chow et al., 2005).

Here we want to claim also that every emotion has a complex functional and semantic dimensionality: For instance, guilt, shame, etc. are feelings of having lost something important and they are combined with a negative self-experience to have done something that socio-culturally is not adequate.

Finally it has to be mentioned that so called “intentional content” of emotions is often referred to as the result of unsatisfied desires: the experience (feeling) of strangeness implies the desire for familiarity, the experience of being alone induces the desire for affiliation, etc. This emotion-to-motivation relation will be clarified below.

#### Dynamics of Emotions

If we observe the stimulus-response relation of anxiety or sadness or aggression we might find different shapes of onset and offset of these emotions (dynamics): fast onset is characteristic for anxiety and aggression, but both are slowly declining, depending partially on the subsequent significant stimuli and of course on parallel cognitive processes of coping performance. Sadness might develop a little slower and also can last longer and can flash up if cognitive events that are related to the specific stimulus pop up again (**Figure 10**).

Although we don’t have enough quantitative data about this specificity of these dynamic aspects of emotions (Frank et al., 2017; Kockler et al., 2017), we have to admit that they are relevant in everyday life and thus could be relevant for the constitution of dysfunctional mental dispositions: a chronic psychophysical stress state is one factor for schizophrenic episodes (Ciompi, 2015).

#### Opposing Interactions of Emotions

The principle of opposing mechanisms in the mind is important: psychophysics of color perception shows that three separated but coupled and opposing mechanisms – black-white, green-red, blue-yellow – can explain a wide range of perceptual phenomena like contrast enhancement, after-images, etc. In line with this, the idea of opposing emotions can be developed (James and Allport, 1985; Plutchik, 2002). Already everyday experience shows that pain relievers can induce happiness if pain is away. However, the most confirmed dynamics of interaction of emotions is the pleasure-unpleasure interaction in addiction: after a drug-induced “high” (a-process) a down-state comes up (b-process) and after repetitive consumption periods the level of maximal pleasure is decreasing little by little (drug tolerance). In consequence the application of the drug mainly reduces negative emotional states. This adaptation process by slow down-regulation of emotions is well known and was captured by Richard Solomon with the concept of “allostasis” (Solomon, 1980).

In consequence, we assume basically that positive emotions are antagonized by negative emotions and vice versa.

#### A Network View of Emotions

In line with such a principle of the oppositional organization of emotions, we have to assume a multilateral reciprocal inhibition of emotions: everyday experience shows that if anxiety occurs, aggression-inducing stimuli will evoke a lower anger response, and probably a rapid cycling between these states can occur. Aggression can be reduced by anxiety and depression can be reduced by aggression, etc. Also co-activation can occur in such networks by serial double inhibition, which is not considered here. However, it should be mentioned here, that networks with reciprocal inhibition can develop a slow change in network background activation depending on the sequence of incoming stimuli that are negative or positive regarding to the respective expectations. Further systemic emotion research should explore these dynamics in order to explain sustained unconscious generic processes of possible symptom production, for instance similar to how PA is assuming it (Freud, 1936). Also neurobiology supports this kind of research as it is shown that the amygdala is not only involved in depression but also in addiction and that nucleus accumbens is involved not only in addiction but also in depression (Koob et al., 2014).

### Motives, Needs and Desires

The second group of drivers of the mental processes are motives, needs, motivations, etc. They are distinct mental conditions, with features of experiencing a pressure and a tendency to act. They are more or less explicitly a goal-oriented state of activation. As already mentioned, the content of motives (e.g., desire for intimacy) are emotionally loaded experiences (e.g., feeling of strangeness and isolation). Although escalatory state trajectories of drives are known (e.g., sexual drive) the desires disappear if these goals are obtained. Terminologically, there are distinctions and equivalencies of terms like motives, drives, needs, desires, will, etc. Here we use the term motives and desires in an equivalent meaning in order to summarize these goal-directed activations of behavior. Similar to emotions, a nearly endless number of motives can be found in psychological literature: Nearly for every object in the world where an individual develops an affinity to can be called a “desire (or need) for x”: I need new shoes, new pants, a new parking license for the car, etc. Regarding a taxonomy of motives, 2, 3, or 6 or dozens of main (or primary) motives are distinguished. For instance, Freud at some periods of his research (e.g., 1920) used two opposing drives – Eros and Thanatos (Freud, 2003), Abraham Maslow (Maslow et al., 1987) used 6 and more needs (physiological needs, security desire, binding desire, etc.), the clinical psychotherapy researcher Klaus Grawe suggested 4 basic needs (Grawe, 2004): orientation and control, social appreciation, pleasure maximization, binding and self-value maximization.

#### Conflicts as Antagonisms Between Motives

Certain configurations of motivations that converge on the same goal but with opposing behavior or opposing motivations appear as “conflicts” or inconsistencies (Grawe, 2004): autonomy quest vs. dependency quest, approach-avoidance conflicts as incongruencies or regarding internal conflicts like id-superego conflicts that indicate discordance (intrapsychic conflicts).

This is in line with the concept of an antagonistic organization of motives as observations of everyday behavior and psychoanalytic literature show: the drive for autonomy opposes the need of dependence, the desire for supply counteracts the demand for self-sufficiency, the desire to subordinate is counterbalanced by the desire to dominate, the need to accept a real negative self-related information collides with the self-idealization (or the intended positive self-image), all these drives determine the mental balance. One multi-axial taxonomy of conflicts is supposed by the working group on Operationalized Psychodynamic Diagnostics (OPD, Arbeitskreis OPD, 2009), focusing conflicts on conflict-axis 3 (e.g., Individuation versus dependence) or on axis 4 (Structure-axis: e.g., Identity(dis)integration) or on axis 2 (Relationship-axis: e.g., submission versus control). Nevertheless, the specific axes remain unrelated to each other. For clinical decisions, for example the topic Self value conflict (S.-ideal / S.-real) could be seen as a problem on Axis 4, but in fact the leading symptom might be the accompanying affect like aggression.

#### Integration of Emotion and Motivation

**FIGURE 12:**
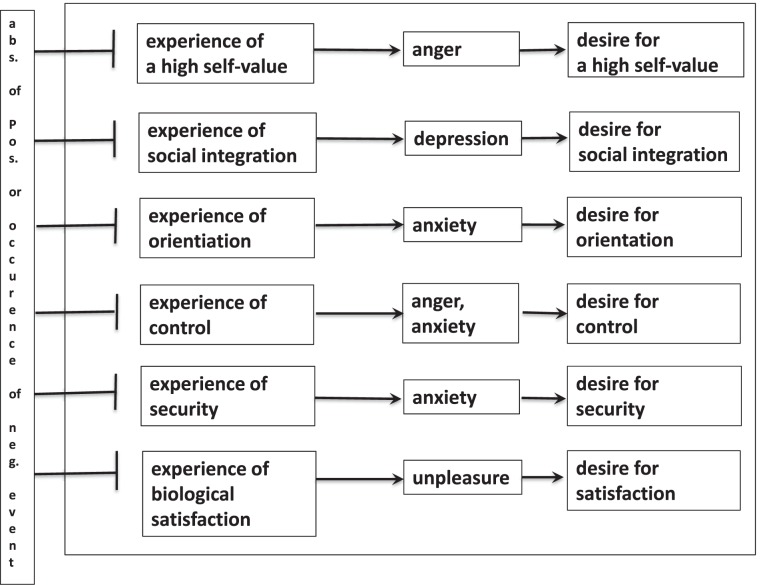
Hypothetical structure of conscious experience, composed by the list of basic needs of Grawe. In this situation they are not satisfied by occurrence of contra-productive stimuli that evoke basic emotions that in turn induce (or accompany) upcoming desires to experience satisfaction of the respective desires.

Here we use the basic and universal term “experience” to describe the subjectivity of conscious emotions and desires. In this view, everyday experience can be conceptualized as an interplay of sensory and motor processes (including cognition) with affective-motivational processes (mechanisms; see **Figure 12**). Some examples:

- Perception of a negative ascription like “you are a loser” is related to an experience of a degraded self and could evoke a state of anger and the consecutive desire for self-affirmation.
- Experience of social ignorance (or non-response to social signals that were addressed to another person) or loss of an important other, leads to the experience of self-devaluation and also sadness, that under chronic condition can lead to a depressive symptomatology that in turn induces a desire for social support.
- Unexpected threatening events can induce anxiety that evokes the desire for security.

**FIGURE 13:**
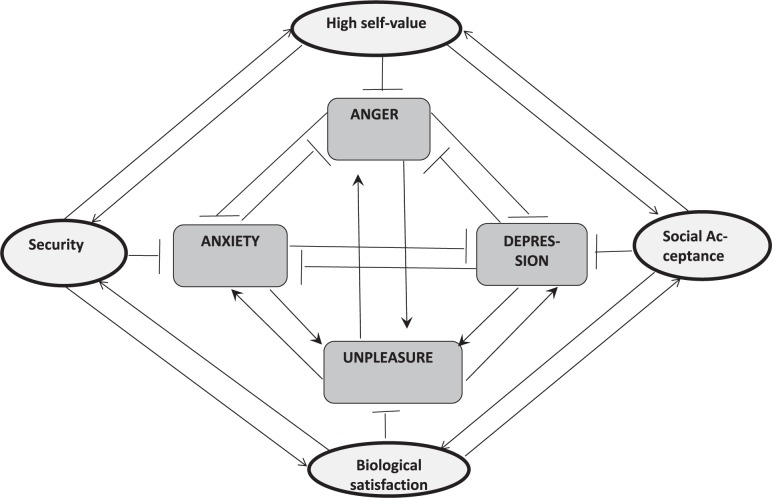
Model of systems complex of the desire system coupled with the emotion system. Bear in mind: the component “pleasure” of **Figure 11** is substituted here by the opposing system “unpleasure”.

From a phenomenological view, all these reactive emotions evoke the desire to experience a higher self-value, more social acceptance and/or more security. Unpleasing levels of satisfaction of biological needs enforce the respective emotional states and the other way round: these emotional states can evoke compensatory activities that satisfy biological needs such as eating excessively (binge eating) or consuming psychotropic drugs in order to reduce “tension”. This tension can be interpreted as the result of the experienced difference of expected stimulation as a set point and the experienced stimulation as an actual value within a control loop of action regulation as it was mentioned before s (see **Figure 13**).

### Systemic Input–Output View

Taking all together about emotions and motives we suggest to conceptualize an affective-motivational systems complex consisting of the network of emotions together with a network of desires that are coupled in multiple ways: the desires are connected mainly in a reciprocally activating way with the tendency to escalate by this interaction, whereas the emotion network has the tendency to regulate itself downward by reciprocal inhibition, except the connections with the subsystem “unpleasure” that is also reciprocally activating the negative emotions. This down-regulation in the network can be seen as equivalent to a subthreshold activity that phenomenologically matches to the concept of unconsiousness.

The essence of this qualitative systemic model of the system complex of emotive-motivational systems regards the potential for modulatory self-organization of activity in an activity landscape of nodes that represent distinct experiential qualities that in sum produce a multifaceted experience of mixed emotions and desires. As a superimposed macro-driver the cognitive system could operate by phantasies as simulations of the self and the environment. It could operate not only as a driver but also as a brake of these dynamic processes.

### A Computational Model of Social Attachment and Avoidance – The Zurich Model

**FIGURE 14:**
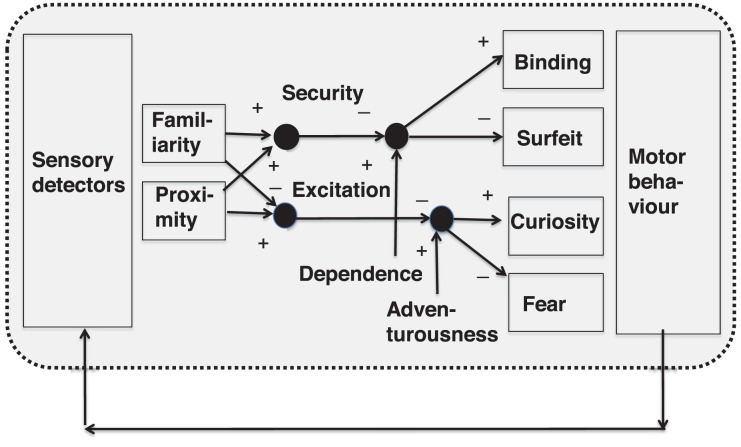
Explanatory circuit model of the latent causal structure of different behavior patterns, by integrative processing of proximity and familiarity of an external object that determines binding behavior and fear and other behavior dispositions.

It is well known that several motivational conflicts have to be resolved during early development like binding and autonomy. This dynamics was captured by cyber-systemic psychologist Norbert Bischof in a complex computational control loop model that explicates the functional connectivity of internal states and drives and their behavioral consequences (Bischof, 1975; **Figure 14**):

- If an external object (e.g., another person) comes near to a person and if it is familiar to the person, an experience of high security arises. This experience is subtracted from the experienced level of dependence: if security is high but dependence is higher, the subtraction results in a positive value and in this case binding behavior occurs. If the subtraction result is negative, surfeit occurs.
- If proximity to an object is high and if familiarity is low, a high excitation emerges. This excitation is subtracted from the desire to have an adventurous experience. If this desire is low but excitation is high, the result is negative and therefore fear arises.

This model shows that even by semi-quantitative explorations a more precise functional understanding of mental processing is possible.

### Perspectives

The intrinsic dynamics of emotions and of conflicts of desires could be highlighted by a systemic re-conceptualization. Published computational models such as the Zurich model of attachment could be extended systematically, quantified and explored by computer simulations for heuristic purposes.

## An Integrative but Differentiated Framework for Psychology/ Psychopathology

These short spotlights to specialized fields of the sciences of the mind should indicate a converging roadmap for the development of a systemic psychology and systemic psychopathology. As we have figured out initially, systemic psychology has to be based on a repertoire of concepts of elements of the mind (e.g., perception, thinking, memory) that are supposed to interact with each other in an activating and inhibiting mode. Regarding this, we criticize briefly the RDoC as a current research-oriented taxonomy of mental disorders, and then we re-consider the continental view of psychopathology and corresponding academic psychology.

### The Matrix of Research Domain Criteria

As already mentioned in the first section, RDoC subdivides the mental functions of the brain into 5 specialized subsystems (Insel et al., 2010): systems for arousal/sleep, negative valence, positive valence, cognition and social processes. These categories are again subdivided into several subconstructs such as working memory, etc. Research on these issues should be conducted with methods of self-reports, behavior, experimental paradigms and by neurobiological methods that explore different levels of neuronal organization. The systems’ mental functions fit partially to the singular functions that are checked in context of classical psychopathology. However, RDoC does not aim to substitute classical psychopathology or replace diagnoses, it should be used for research purposes only. NIMH states: “…RDoC is an experiment to determine if a diagnostic approach based on biology, behavior, and context will be useful for mental disorders”(NIMH, 2017). Finally it has to be mentioned that RDoC is a two-dimensional list but not a reference model and it is not framed by any theoretical concept of the psycho-sciences. Regarding the valence systems that are constructs for emotional and motivational states together, some authors already suggest essential extensions of the domains by integrating the new domain emotion regulation (Fernandez et al., 2016).

Finally it should be minded that the term “system” mainly means a neuronal level of organization as a reference object of behavioral operation. With these critical thoughts, we go back to classical clinical psychopathology.

### A Structured Systems View of the Mind – The Skeleton of the Mental System

As it was mentioned already, any system and also the mental system must defined by its elements, and its relations. The state of the system and its components – usually in case of pathology an over-activity or under-activity (or activation) has to be specified. Such descriptive-classificatory categories are represented in the AMDP scheme of clinical psychiatric examination. They should be an everyday tool for documentation of the mental function as they appear to the trained psychiatrist at the beginning of the contact and as a summarizing procedure when psychiatric history taking and psychiatric exploration are finished (Broome et al., 2017). In consequence, we have a list of functions and dysfunctions (symptoms) that describe the level of mental functionality regarding clinical issues such as syndromes and diseases (**Table 1**).

**Table 1:** Concepts of mental operations and operators and some disorders.

Term	Interpretation	Pathology
Perception	Uptake of information	*Hallucinations*
Expectation	Expectation of event	*Obsessive mood*
Thinking and thoughts	Mostly bound and expressed by words and phrases	*Thought disorders*
Memory	Storage and recall of information	*Memory disorders*
Emotions	Emotions, feelings (anger, fear or grief)	*Affective disorders*
Motives	Drives, desires, needs	*Abulia*
Plans	Intentions to act to achieve a situation or personal state	*Delusional action plans*
Behavioral programs	Motor pattern that are related to action	*Behavioral disorders*
Personality	Transsituational invariant behavioral disposition; typical affective- motivational characteristics of a person	*Personality disorder*
Self	Core area of the mental, largely unconscious	*“Split self”*
Ego	Conscious portion of the (operational) self	*Ego disorders*
Self-image (self concept/model)	Partly conscious scheme of the person about him/herself	*Unstable, negative or positive self-image*
Environmental image (concept-model of the external world)	Image of the mainly surrounding world of the person, the social environment such as the family, friends, etc.	*(delusional) Image of the world*

**FIGURE 15:**
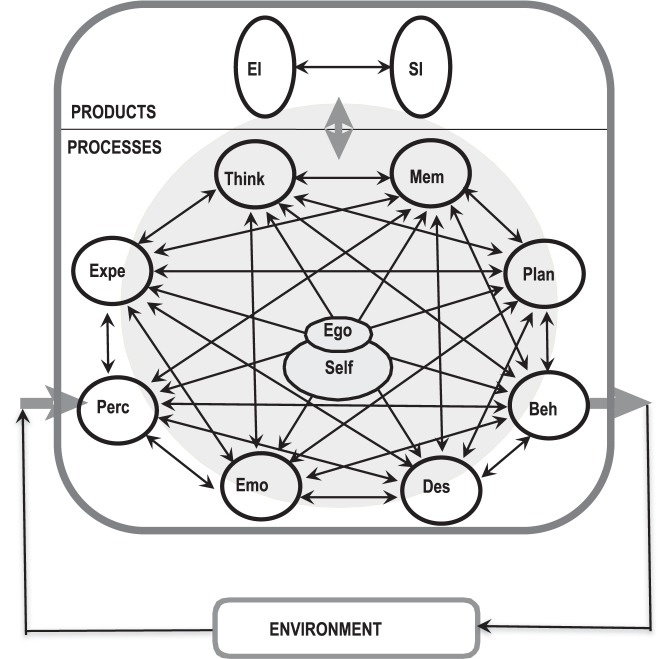
The list of mental operators (subsystems) as an operationally closed network. Consciousness is represented here as a shaded circle, regarding the metaphor of light and dark. Not all relations – especially of the ego/self – are depicted here. Perc, perception; Expe, expectation; Thin, thinking; Mem, memory; Plan, plans of behavior; Beh, behavior; Des, desires; Emo, Emotions; EI, environmental image; SI, self-image as affective-cognitive schemata.

This list should be converted to a network model of the mind and its contents such as the representations of the self, the environment and their interrelations (**Figure 15**). By this model, any mental event, e.g., a state of anxiety, can be explored analytically regarding its influences on perception, expectation, thinking, memory, planning of behavior, behavior and motivations to act or not to act. Also our initial example of addictive behavior can be explored theoretically by pathways of thinking and emotion, as well as being grounded on a labile self image/self. This multi-effectual view of a single mental operator (e.g., one emotion) corresponds to the view of affect-logic by Luc Ciompi, who proposes such dispersed network effects of persisting anxiety, anger and sadness as causes of mental disorders such as schizophrenia (Ciompi and Schneider, 1988; Ciompi, 1997). Finally, it is obvious that this network-concept corresponds quite well with neurobiology as it was mentioned in the introduction referring to addiction (Koob et al., 2014).

### Perspectives

Considering the micro-level of the mind, by up-scaling a macroscopic view can be sketched regarding the global circuitry of mental processes and their interaction with affective-cognitive schemata. For future research the vertical top-down differentiation of models of mental processing should be developed systematically.

## Conclusion

A more precise understanding of terms like “structure” and “dynamics” as they are used in the clinical psycho-sciences seems to be possible if they are related explicitly to systems science. In line with this, it should be kept in mind that the use of systemic concepts should exceed pure metaphors. A comprehensive use of systemic concepts and methods and theories in psychology needs a network-oriented reconstruction of the pool of psychological concepts. This was demonstrated here for some examples. Finally, a global conceptual model should be developed that explicates a differentiated reference model of the mental.

For the future, we think that a working group or network of corresponding theoretical researchers across different psycho-disciplines in cooperation with experts in systemic modeling could proceed in integrating various psychological conceptions of the mind. For instance, it is important to define the components, their relations, their state variables, etc. as precisely as possible. In a next step, these terms can be used as bridge-concepts if they enable the connection of different psychological perspectives more closely (Haken and Tschacher, 2017). Empirical research alone – also in the network perspective – might not be sufficient for a better theoretical understanding of psychological mechanisms of mental disorders. A crucial role might come to psychoanalysts who are interested in systems science as PA offers the richest pool of theoretical concepts based on neurobiology, social science, clinical observations and systems science that provides an integrative “meta-framework” (Galatzer-Levy, 1995; Seligman, 2005).

## Author Contributions

HL-S conceived psychoanalysis as an integrative systems psychology and discussed psychoanalytic and psychiatric theories. FT discussed systems science as well as the basic steps for development of systems psychology. HL-S and FT integrated the concepts stepwise in iterative discussions of the paper.

## Conflict of Interest Statement

The authors declare that the research was conducted in the absence of any commercial or financial relationships that could be construed as a potential conflict of interest.
